# Integrating Biological Advances Into the Clinical Management of Breast Cancer Related Lymphedema

**DOI:** 10.3389/fonc.2020.00422

**Published:** 2020-04-02

**Authors:** Marco Invernizzi, Gianluca Lopez, Anna Michelotti, Konstantinos Venetis, Elham Sajjadi, Leticia De Mattos-Arruda, Michele Ghidini, Letterio Runza, Alessandro de Sire, Renzo Boldorini, Nicola Fusco

**Affiliations:** ^1^Physical and Rehabilitative Medicine, Department of Health Sciences, University of Eastern Piedmont “A. Avogadro”, Novara, Italy; ^2^School of Pathology, University of Milan, Milan, Italy; ^3^Division of Pathology, Fondazione IRCCS Ca' Granda, Ospedale Maggiore Policlinico, Milan, Italy; ^4^Ph.D. Program in Translational Medicine, University of Milan, Milan, Italy; ^5^Divison of Pathology, IRCCS European Institute of Oncology (IEO), Milan, Italy; ^6^IrsiCaixa Foudation, Hospital Universitari Germans Trias i Pujol, Badalona, Spain; ^7^Division of Medical Oncology, Fondazione IRCCS Ca' Granda, Ospedale Maggiore Policlinico, Milan, Italy; ^8^Rehabilitation Unit, “Mons. L. Novarese” Hospital, Moncrivello, Italy; ^9^Pathology Unit, Department of Health Sciences, Novara Medical School, Novara, Italy; ^10^Department of Oncology and Hemato-Oncology, University of Milan, Milan, Italy

**Keywords:** breast cancer related lymphedema, pathobiology, genetics, breast cancer, survivorship, quality of life

## Abstract

Breast cancer-related lymphedema (BCRL) occurs in a significant number of breast cancer survivors as a consequence of the axillary lymphatics' impairment after therapy (mainly axillary surgery and irradiation). Despite the recent achievements in the clinical management of these patients, BCRL is often diagnosed at its occurrence. In most cases, it remains a progressive and irreversible condition, with dramatic consequences in terms of quality of life and on sanitary costs. There are still no validated pre-surgical strategies to identify individuals that harbor an increased risk of BCRL. However, clinical, therapeutic, and tumor-specific traits are recurrent in these patients. Over the past few years, many studies have unraveled the complexity of the molecular and transcriptional events leading to the lymphatic system ontogenesis. Additionally, molecular insights are coming from the study of the germline alterations involved at variable levels in BCRL models. Regrettably, there is a substantial lack of predictive biomarkers for BCRL, given that our knowledge of its molecular milieu remains extremely puzzled. The purposes of this review were (i) to outline the biology underpinning the ontogenesis of the lymphatic system; (ii) to assess the current state of knowledge of the molecular alterations that can be involved in BCRL pathogenesis and progression; (iii) to discuss the present and short-term future perspectives in biomarker-based patients' risk stratification; and (iv) to provide practical information that can be employed to improve the quality of life of these patients.

## Introduction

Breast cancer-related lymphedema (BCRL) is a particular form of secondary lymphedema occurring after axillary surgical procedures and/or irradiation in 14–54% of breast cancer survivors (1). Its clinical signs are related to an augmented volume of the upper limb due to tissue swelling and subsequent fibrosis (2). These include impaired function and strength, malaise, pain, comorbidities, and psychosocial frailty (3, 4). The diagnosis of BCRL is established by the measurement of the arm volume. Over the past decades, a wide variety of strategies have been proposed to identify and quantify alterations in the upper limb volume, including tape, perometry, bioimpedance, imaging (e.g., lymphography and magnetic resonance imaging), and augmented reality tools (5–9). BCRL prevention is centered on general healthcare suggestions, such as physical activity, body weight control, skincare, avoidance of infections (10). However, microsurgery-based primary prevention schemes, such as axillary reverse mapping and lymphatic-venous bypass, are showing promising results (11). For decades BCRL has been considered as an incurable condition but several therapeutic approaches are now available, both in the setting of physical therapy (e.g., complex decongestive therapy, manual lymph drainage, Qigong exercise, yoga, laser therapy, extracorporeal, shock wave therapy) and surgery (e.g., tissue excision, derivative microsurgery, microsurgical reconstruction, vascularized lymph node transfer, block of sympathetic innervation) (8, 12–15). Regrettably, the pre-surgical identification of high-risk individuals is extremely challenging.

Despite these insights, the multifaceted biology of BCRL remains poorly understood due to the substantial lack of molecular data. Therefore, tailored prevention and treatment schemes are not routinely performed in these patients. In this review article, we seek to outline the biological and genetic changes in the lymphatic system development and impairment in breast cancer survivors, focusing on possible biomarkers for its risk assessment, diagnosis, prognostication, and treatment.

## Pathophysiology

### Ontogenesis of the Lymphatic System

**Figure 1:**
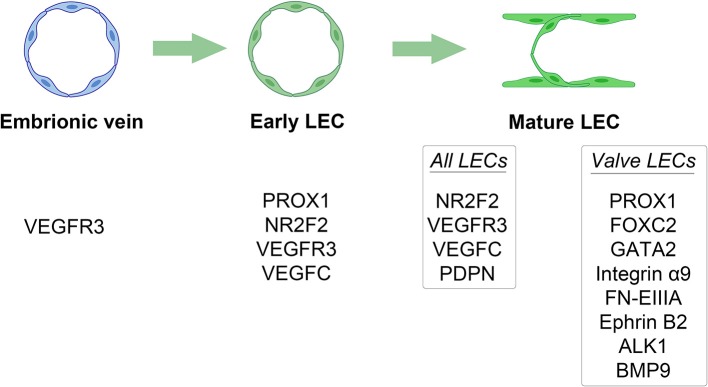
Key molecular and transcriptional events in the lymphatic system ontogenesis. Different stages of lymphatic system development are outlined by their distinct stage-specific expression of different molecules. LEC, lymphatic endothelial cell.

The lymphatic system is composed of a complex network of vessels and organs complementary to the cardiovascular system (16). It plays a crucial role in several biological events, including immune response and homeostasis of interstitial fluids, cells, molecules, and tissue debris (17, 18). At early stages of embryogenesis, the lymphatic vessels develop from the embryonic veins through the stepwise expression of numerous molecules, including prospero-related homeobox domain 1 (PROX1) and nuclear receptor subfamily 2, group F, member 2 (NR2F2) (17, 19). Interestingly, the silencing of these two genes in mice prevents lymphangiogenesis (20, 21). The lymphatic sac, which is lined by lymphatic endothelial cells (LECs), represents the earliest lymphatic structure (22). The LECs express lymphatic-specific proteins, such as vascular endothelial growth factor C (*VEGFC*). The absence of this molecule in animal models results in diffuse and lethal tissue swelling (23). The separation of the lymphatic system from the blood vessels leads to the formation of the lymphatic plexus (24). This process is mediated by a signaling pathway in which podoplanin (PDPN), expressed by the LECs, interacts with its receptor on platelets, promoting their aggregation (25). Subsequently, platelet microthrombi form a physical barrier that interrupts the communication between lymphatic and blood vessels (26, 27). Inactivating mutations in *PDPN* are related to defects in vascular system separation, and subsequent abnormal shunts (24, 27, 28). The development of a contractile component (i.e., myoepithelial cells) coupled with that of a valve system allows for the unidirectional flow of the lymph fluid. This phase is characterized by the differential expression of PROX1, forkhead box protein C2 (FOXC2), GATA2, integrin α9 (ITGA9), and its ligand extra domain A fibronectin (29, 30). Their deficiency is associated with failure in valve formation and consequent lymphedema (31–33). The key molecular and transcriptional events in the lymphatic system ontogenesis are outlined in Figure 1.

### Fluid Drainage and Anatomic Considerations

**Figure 2:**
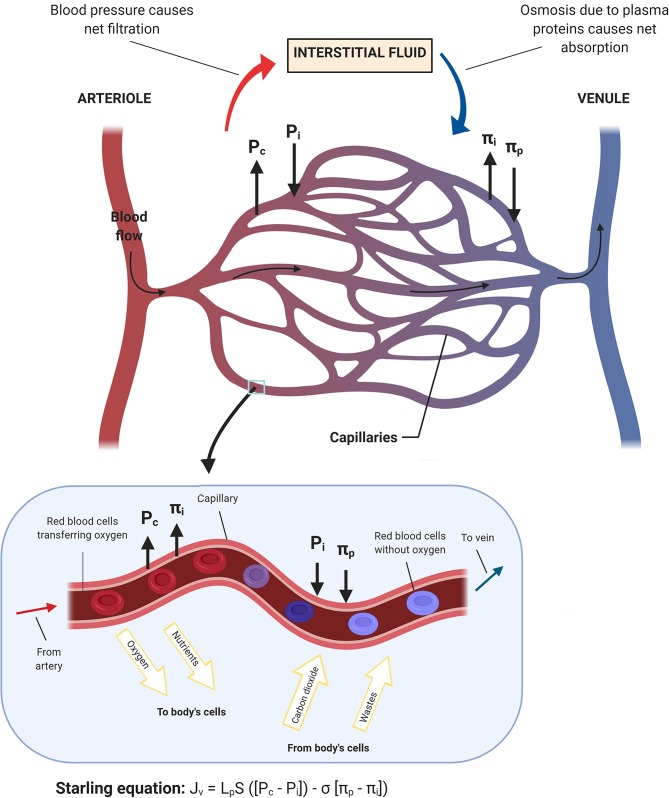
Schematic representation of the fluid homeostasis based on the Starling equation. When the blood flow goes into the capillary, the capillary hydrostatic pressure (Pc) and the interstitial oncotic pressure (πi) drive oxygen and nutrients toward body's cells. Conversely, when blood moves toward venules, the interstitial fluid hydrostatic pressure (Pi) along with the plasma oncotic pressure (πp), which are mainly applied by the surrounding proteins, drive wastes and carbon dioxide into the capillary and subsequently out of the body.

**Figure 3:**
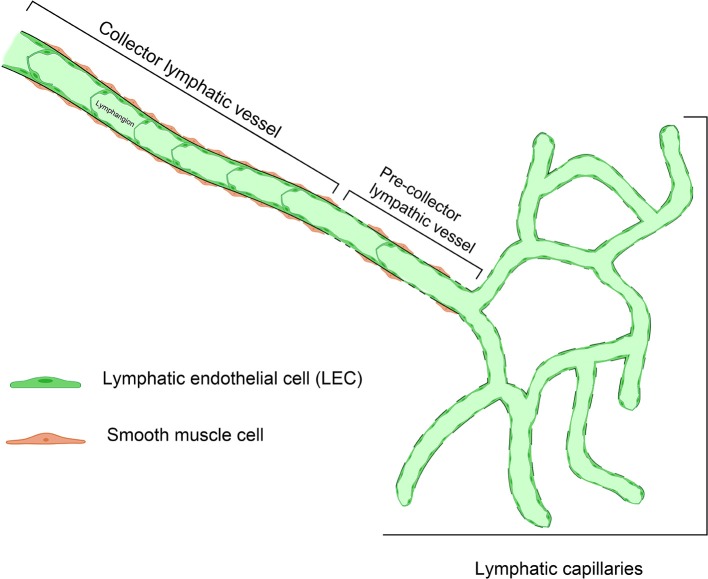
Representative structure of the different types of lymphatic vessels. Small, branching lymphatic capillaries lined by a single file of lymphatic endothelial cells (LEC) are connected to pre-collector lymphatic vessels, showing tracts with a discontinuous basal lamina. The collecting vessels, whose functional unit is the lymphangion, are larger in diameter and have a prevalent propulsion function.

The lymph flow is determined by both intrinsic and extrinsic forces that promote lymph propulsion in the lymphatic conduct; intraluminal one-way valves minimize the backflow (34). Given the lack of a central pump for the lymph fluid, the flow is driven by rhythmic contractions of smooth muscle cells in the lymphatic vessels (35). Arterial pulsations, skeletal muscle compression, fluctuations of central venous pressure, gastrointestinal peristalsis, and respiration are also involved in this mechanism, representing the passive lymph pump. The entire interstitial drainage process is governed by the Starling equation (Figure 2). Three types of lymphatic channels are present, namely capillaries (also referred to as initial lymphatics), pre-collecting vessels, and collecting vessels (Figure 3). Capillaries are blind-ending vessels composed of a single layer of non-fenestrated LECs, with an incomplete basal lamina. These structures have specialized junctions and anchoring systems that act synergistically in promoting the passage of lymph from the interstitium to the lumen (36). Pre-collecting vessels are characterized by the alternation of propulsion segments (i.e., provided with muscular coat and intraluminal valves) and tracts with an absorbing architecture (i.e., irregularly-arranged of smooth muscle cells and discontinuous basal lamina) (37). These vessels converge into the collecting vessels, whose functional unit is represented by the lymphangion, defined as the segment between two valves (38). Lymphangions have zipper-like junctions between LECs, continuous basement membrane, well-represented muscular layer, and bi-leaflets one-way valves (39). It should be noted that the lymphatic network is asymmetric. Hence, the right lymphatic duct, which drains in the right subclavian vein, is present only in the right upper limb, the right side of the trunk, and the head and neck region (40), while all other territories are drained by the thoracic duct into the left subclavian vein (41).

### Understanding the Tissue Milieu: Inflammation and Matrix Response

The soft tissue composition is a key factor in lymphatic homeostasis, as demonstrated by the increased risk of lymphedema related to fat accumulation (8, 42, 43). Importantly, the lymphatic fluid stasis regulates the expression of genes with regulatory functions in adipogenesis, such as peroxisome proliferator-activated receptor gamma (*PPARG*) and CCAAT/enhancer-binding protein alpha (*CEBPA*) (44). Another key factor is represented by the adiponectin, a protein hormone involved fatty acid breakdown, that contributes to the signaling between adipose and immune cells and regulates the chronic inflammatory response (44). This protein can be overexpressed in response to lymphatic fluid stasis, thus mediating the tolerance to proinflammatory stimuli in the case of obstruction (44, 45). Recently, adipose-derived stem cells co-cultured with human lymphatic endothelial cells have been shown to induce mRNA expression of lymphatic markers and proliferation/migration of lymphatic endothelial cells, without affecting tube formation (46). These data pave the way for possible engineering therapies to improve secondary lymphedema outcome.

Fibrosis and increased subcutaneous adipose tissue volume are the two main aspects of tissue remodeling which characterize late-stage BCRL (47). Therapeutic interventions designed to reduce their presence can increase the lymphatic function (48). In this respect, both cytokines and immune cells promote lymphangiogenesis, with a subsequent potential therapeutic role (49, 50). Interestingly, alternatively activated macrophages (M2) are often increased in lymphedema tissues, particularly in the setting of T helper 2 cell-mediated anti-inflammatory response in fibrotic phases (45). The macrophage infiltration in lymphedema decreases the overall inflammation and inhibits fibrosis (45). It has recently been proposed that a high capillary filtration coefficient coupled with increased plasma levels of VEGFC may constitute important biological traits of BCRL patients (51). Hence, a systemic increase in VEGFC promotes microvascular permeability, and an overload of the remaining lymphatic drainage capacity (52). On the other hand, the recovery of interstitial fluid drainage and the natural resolution of acute BCRL are not hindered by the administration of VEGF receptors blockers, suggesting that these processes are lymphangiogenesis independent. Taken together, the interstitial matrix plays a central role in the increase of lymph drainage (53).

## Risk Stratification: Who is Likely to Develop BCRL?

Despite early detection can improve BCRL patients' outcome, the preventive options available to date are extremely limited (54). The physical disruption of the arm lymphatics, such as in case of axillary lymph node dissection (ALND), is a well-established determinant of BCRL (55). Of note, both the number of lymph nodes removed and the number of metastatic lymph nodes are associated with an increased risk (56, 57). It has been hypothesized that this could be due to the higher dose of radiations that these patients receive in the axilla (55, 57). Hence, radiation-induced necrosis is likely to be involved BCRL pathogenesis (58). A higher prevalence of BCRL has also been observed in patients treated with anti-tumor systemic drugs, such as taxanes and trastuzumab, probably due to diminished lymphatic contractility (59–61). The correlation between body max index (BMI) >25 kg/m^2^, post-operative weight increase, dyslipidemia, and BCRL has been widely demonstrated (8). However, novel tumor-specific pathological features, such as peritumoral lymphovascular invasion and the extra-nodal extension of the metastatic deposits, have recently been proposed to improve BCRL risk stratification (56, 57). In general, there is a wide agreement that breast-conserving surgery is protective against long-term complications, including BCRL (62).

**Figure 4:**
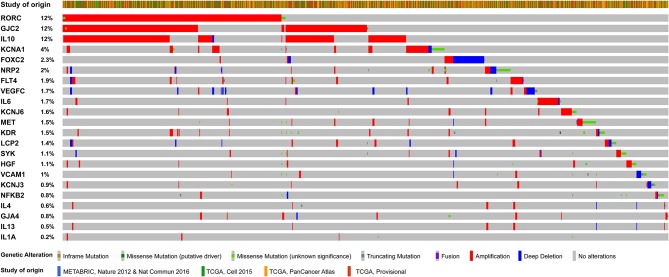
Oncoprint visualization of the somatic molecular alterations in breast cancers (*n* = 3,394 samples) involving 22 genes with reported germline alterations in BCRL patients. Each column represents a sample, each row represents a gene, as reported on the left. The genes were sorted by alterations frequency (percentage on the left). Types of alterations and study of origin (publicly available at cBioportal.com) are color-coded on the basis of the legend on the bottom.

In addition to the classical mechanistic explanation, the study of the genetics underpinning BCRL has provided intriguing insights. Several germline alterations in genes involved at various levels in lymphangiogenesis have been documented in BCRL patients, suggesting a possible role for individual predisposition in the development of lymphedema following breast cancer therapy (Table 1). These genes include lymphocyte cytosolic protein 2 (*LCP2*), spleen associated tyrosine kinase (*SYK*), endothelial cell adhesion proteins (i.e., promoters, growth factors, and their receptors), interleukins, and K-channel genes (50, 63–72). Interestingly, these genes show recurrent somatic alterations in breast cancer, with a higher prevalence of gene copy-number alterations (CNAs) than somatic mutations (Figure 4). Despite these relevant observations, no tumor-specific recurrent molecular alterations have been identified in BCRL patients.

**Table 1:** Genes that have been related to BCRL predisposition.

**Genes**	**Gene family**	**Function**
*LCP2*	Signal-transducing adaptor protein	T-cell activation.
*SYK*	Spleen tyrosine kinase	Adaptive immune receptor signaling; Cell proliferation, differentiation, and phagocytosis; Separation of newly formed lymphatic vessels from the blood vasculature.
*VCAM1*	Cell adhesion promoters	Vascular endothelial cell adhesion and signal transduction.
*HGF, HGFR/MET, VEGFC, FLT4, VEGFR2/KDR, NRP2*	Growth factors and receptors	Mitogenesis and morphogenesis; Embryonic development; Myocardial development; Epithelial-mesenchymal transition; Liver regeneration. Cardiovascular development; Angiogenesis; Lymphangiogenesis; Endothelial cell growth; Permeability of blood vessels.
*NFKB2, RORC, FOXC2*	Transcription factor-coding	Inflammation and immune response Lymphoid organogenesis (in mice). Valves development.
*GJC2, GJA4*	Connexins	Arteriogenesis; Oocyte survival; Oligodendrocyte development.
*IL1A, IL4, IL6, IL10, IL13*	Interleukins	Apoptosis and cell proliferation; Immunoregulation and inflammation; Expressed also in endothelial cells.
*KCNA1, KCNJ3, KCNJ6, KCNK3*	K channel proteins	Electrochemical gradient across cell membranes; In the lymphatic system facilitate lymph flow.

## Genomic Landscape and Molecular Heterogeneity

### Genetic Determinants and Putative Driver Alterations

It has been suggested that BCRL susceptibility might have individual determinants, raising the possibility that therapy-associated lymphatic injuries might heighten a pre-existing deficit in the lymphatic function (73). Hence, among patients with BCRL, those with the involvement of the whole arm and hand showed an impairment of lymphatic function also in the contralateral unaffected arm (74). Following this circumstantial evidence, the detection of recurrent genetic traits is strategic to achieve the goal of precision medicine in BCRL.

#### Lymphangiogenic and Angiogenic Genes

In the last decade, the presence of alterations in genes related to lymphangiogenesis, lymphatic function, and permeability has been unraveled in BCRL. One of the most studied genes is *LCP2*, which is involved in the immune response through the modulation of the T-cell signaling pathway (75). In addition, *LCP2* plays a central role in the lymphatic development, participating in the platelet-dependent mechanism of separation between blood and lymphatic vessels during embryogenesis (26, 76). Alterations in this gene are related to inherited lymphedema (77, 78). Copy-number alterations in *LCP2* occur in 1.4% of breast cancer patients (Figure 4). They show a strong tendency toward co-occurrence with alterations in other genes known to be implicated in BCRL, such as interleukins (i.e., *IL4, IL10, IL13*) and neuropilin 2 (*NRP2*), as detailed in Table 2. NRP2 is a transmembrane glycoprotein expressed in blood and LECs, which is upregulated in the presence of ischemia and/or hypoxia (79–81). This protein is considered an important mediator of angiogenesis and lymphangiogenesis, acting as a co-receptor with VEGFC. This a molecule is encoded by two genes, namely *VEGFC* and Fms-related tyrosine kinase 4 (*FLT4*) (82–84). Somatic alterations in *NRP2, FLT4*, and *VEGFC* have a strong tendency of co-occurrence in breast cancer (Table 2) and may predispose to secondary lymphedema (68, 69, 73). Vascular cell adhesion protein 1 (VCAM1) is an adhesion molecule that promotes lymphocyte trans-endothelial migration in cytokine activated endothelium (85, 86). This adhesion molecule fosters tissue inflammation and contributes to lymphedema progression. CNAs in *VCAM1* occur in ~1% of breast cancer patients (Figure 4) and they are simultaneously present together with somatic alterations in other genes implicated in BCRL pathogenesis (Table 2). These include interleukins, nuclear kappa factor-beta 2 (*NFKB2*), *VEGFR/KDR*, as well as the hepatocyte growth factor (*HGF*) and its receptor *MET*. Six *HGF/MET* mutations in the sites of interaction and binding domain, respectively, were identified in secondary lymphedema, suggesting that altering this pathway can increase individual risk of developing lymphedema after breast surgery and thus providing a new potential therapeutic target (66). Another important gene in BCRL is represented by RAR-related orphan receptor gamma (*RORC*), which is known to be implicated in lymphangiogenesis, lymph node organogenesis, immune response, and cancer (87). Regrettably, the specific functions of this transcription factor in humans remain poorly understood. Interestingly, both somatic missense mutations and gene amplification in *RORC* are highly recurrent in breast cancers, being detected in up to 12% of patients (Figure 4). Alterations in this gene can be observed in patients that harbor alterations in other BCRL genes, such as *FLT4, IL10*, and *VEGFR2/KDR* (Table 2).

**Table 2:** Significant trends in co-occurrence between pairs within genes linked to BCRL in breast cancer public datasets available at cBioPortal.

**A**	**B**	**Neither**	**A not B**	**B not A**	**Both**	**log_**2**_ O.R**.	***p*-value**	***q*-value**
LCP2	FLT4	2756	26	35	14	>3	<0.001	<0.001
LCP2	IL13	2785	34	6	6	>3	<0.001	<0.001
LCP2	IL4	2782	34	9	6	>3	<0.001	<0.001
LCP2	GJC2	2476	27	315	13	1.92	<0.001	0.003
LCP2	IL10	2490	30	301	10	1.463	0.009	0.034
LCP2	NRP2	2737	34	54	6	>3	<0.001	0.001
NRP2	KCNA1	2687	50	84	10	2.678	<0.001	<0.001
NRP2	IL13	2762	57	9	3	>3	0.002	0.01
NRP2	IL4	2759	57	12	3	>3	0.003	0.017
NRP2	KCNJ6	2739	56	32	4	2.612	0.006	0.028
NRP2	KCNJ3	2750	57	21	3	2.785	0.013	0.045
MET	NRP2	2733	38	56	4	2.361	0.011	0.039
VEGFC	NRP2	2727	44	53	7	>3	<0.001	<0.001
VEGFC	FLT4	2739	43	41	8	>3	<0.001	<0.001
VEGFC	RORC	2465	35	315	16	1.839	<0.001	0.001
VEGFC	IL10	2484	36	296	15	1.806	<0.001	0.002
SYK	VCAM1	2774	29	25	3	>3	0.004	0.017
VCAM1	NFKB2	2781	25	22	3	>3	0.002	0.01
VCAM1	GJA4	2784	25	19	3	>3	0.001	0.008
VCAM1	HGF	2774	25	29	3	>3	0.004	0.017
VCAM1	IL13	2793	26	10	2	>3	0.006	0.026
VCAM1	KDR	2763	25	40	3	>3	0.008	0.033
VCAM1	IL4	2790	26	13	2	>3	0.009	0.034
VCAM1	MET	2766	23	37	5	>3	<0.001	<0.001
MET	KDR	2753	35	36	7	>3	<0.001	<0.001
MET	KCNA1	2700	37	89	5	2.035	0.012	0.04
HGF	MET	2763	26	36	6	>3	<0.001	<0.001
SYK	MET	2760	29	39	3	2.872	0.011	0.039
RORC	GJC2	2377	126	123	205	>3	<0.001	<0.001
RORC	IL10	2372	148	128	183	>3	<0.001	<0.001
RORC	KCNA1	2432	305	68	26	1.608	<0.001	<0.001
RORC	GJA4	2485	324	15	7	1.84	0.01	0.036
KDR	RORC	2470	30	318	13	1.751	<0.001	0.006
FLT4	RORC	2467	33	315	16	1.925	<0.001	<0.001
NFKB2	GJA4	2787	22	19	3	>3	<0.001	0.006
NFKB2	IL10	2502	18	304	7	1.678	0.015	0.049
GJA4	IL10	2505	15	304	7	1.943	0.007	0.03
FLT4	GJA4	2763	46	19	3	>3	0.006	0.026
GJC2	IL10	2423	97	80	231	>3	<0.001	<0.001
FLT4	GJC2	2475	28	307	21	2.596	<0.001	<0.001

#### Immunomodulation and Inflammatory Response

Variations in pro-inflammatory (e.g., *IL1, IL2, IL8, IL17, NFKB2*) and anti-inflammatory (e.g., *IL4, IL10, IL13*) cytokines have been found in the circulating DNA of patients with BCRL (50). Among these, the single nucleotide polymorphisms (SNPs) significantly related to the development of unilateral arm swelling are those targeting *NFKB2, IL10*, and *IL4*. In particular, NFKB2 is a transcription factor involved in a multitude of biological processes, including (but not limited to) angiogenesis, cell proliferation, inflammation, tumorigenesis, and tumor progression (88). Alterations in this gene are relatively rare (~0.8%) in breast cancers and display the strong propensity toward co-occurrence with those targeting IL10, that are highly recurrent (12%), as shown in Figure 4 and Table 2. IL10 is an anti-inflammatory cytokine that acts downregulating the expression of Th1 cytokines, MHC class II antigen-presenting molecules, and costimulatory molecules on macrophages (48). In particular, IL10 influences active transcription factor binding sites that are involved in lymphangiogenesis. Most importantly, this interleukin induces immunosuppression and tumor escape from immune surveillance, particularly in breast cancers lacking the expression of the estrogen receptor (89). Alterations in *IL4* have also been detected in the circulating DNA of BCRL patients. This pleiotropic cytokine is produced by CD4+ T-cells and it has an important role in B-cell immune response modulation (48). This pathway is thought to be involved in alterations observed in lymphedematous tissues, such as fibrosis, adipose deposition, and lymphatic dysfunction (48, 90). Interestingly, it has been recently observed that cyclooxygenase (COX)2 and its product prostaglandin (PG)E2 are overexpressed in breast cancer stroma, having a possible role in lymphangiogenesis and metastatic spread s through lymphatics (91). Specifically, PGE2 activates the EP4 receptor in cancer cells and macrophages, promoting local VEGF-C/D overexpression, and LECs proliferation (91). All this information opens new avenues in BCRL risk stratification, providing that further prospective clinical studies will be designed to investigate whether NFKB2, IL10, IL4, and EP4 can be employed as circulating biomarkers for pre-surgical risk assessment.

#### Transmembrane Diffusion and Inter-cellular Communication

Connexins are a family of specialized transmembrane proteins that form the gap junctions between cells (70). They are crucial for both blood and lymphatic vessel homeostasis (70). Many authors have suggested that connexins may be implicated in the initial development of the lymphatic system, particularly in the formation of the lymphatic valves and sac (92, 93). Mutations in genes encoding the connexins 47 and 37, namely gap junction protein gamma 2 (*GJC2*) and gap junction protein alpha 4 (*GJA4*), have been linked to both primary and secondary lymphedema (67, 72, 94). Intriguingly, *GJC2* CNAs are highly recurrent in breast cancer, being present in 12% of cases in the cBioPortal, as depicted in Figure 4. Furthermore, CNAs in *GJC2* and *GJA4* are significantly present together with somatic alterations in other BCRL genes, such as *RORC, IL10*, and *FLT4* (Table 2). So far, these gap junction proteins represent promising biomarkers in both breast cancer and BCRL prognostication.

#### Membrane Action Potential and Smooth Cell Contraction

Several potassium channel genes were found to be the target of SNPs in the setting of secondary lymphedema. These genes include potassium voltage-gated channel subfamilies A member 1 (*KCNA1*), J member 3 (*KCNJ3*), 2 (*KCNJ6*), and K member 3 (*KCNK3*) (71). In particular, KCNA1 is a transmembrane protein selective for potassium-positive ions; its functions are to shape the action potential and promote the return of the depolarized membrane to its resting state. KCJN3 and KCJN6 are inward rectifying channels that act in an opposite way to voltage gated-channels, supporting the flow of positively charged potassium ions into the cell and stabilizing the resting membrane of cells (38, 71). Finally, KCNK3 is another relevant tissue factor that contributes to the maintenance of the resting potential, giving rise to the background or outward leak potassium-positive currents (38). Despite the great efforts that have been made to determine the influence of genetic predisposition in BCRL pathophysiology, these analyses have several limitations. Larger sample sizes could reveal additional associations between polymorphisms and BCRL.

### Biological Characteristics of the Primary Tumors

The possible existence of molecular indicators evaluable in a pre-operative/operative setting remains one of the key topics surrounding BCRL. For this aim, a search on the public genomic database cBioPortal has been conducted to determine whether genetic alterations associated with both congenital and postsurgical lymphedema occurred also in breast cancer. A correlation between lymphedema candidate genes and mutations in the primary tumor could be useful as an indicator of patients' individual susceptibility, along with the well-known treatment-related risk factors. A query was submitted in order to search genetic alterations of literature driven genes in 2,509 breast cancer samples from METABRIC (Molecular Taxonomy of Breast Cancer International Consortium) project. Notably, in almost all cases genetic alterations found in candidate gene consist of gene amplification, while previous genetic studies individuated single nucleotide polymorphisms associated with BCRL. Most genes were altered in a small percentage of tumor samples, ranging from 0.1 to 2.5%. However, three of them were amplified in at least one-fifth of the breast cancer cases. Specifically, the RORC gene was amplified in 20%, GJC2 in 24% and IL10 in 25% of samples.

To date, the function of RORC's encoded protein in humans remains poorly understood. However, there are several lines of evidence to suggest that this gene may play a part in lymphoid organogenesis and thymopoiesis regulation (87). In addition, RORC protein plays a role in the expression of some clock genes and its expression has been linked to breast cancer survival outcomes (95). RORC overexpression seems to increase distant metastasis-free survival in breast cancer patients (96–98). However, given the lack of knowledge on its precise function and interactions in humans, it is not possible to speculate on the role of RORC in BCRL pathogenesis, preventing also any consideration of the correlation between its amplification and lymphedema occurrence. Connexins are widely expressed in the normal mammary glands, where gap junctions have distinct functions in development and homeostasis, such as modulation of cell proliferation and lactation (99). In advanced breast neoplasms, they are believed to increase the capacity of tumor cells to metastasize through enhancing their invasion and adhesion ability as well as by protecting tumor cells from hypoxia-induced death (100–102). Furthermore, some subtypes of connexins, namely Cx26, Cx32, and Cx43 are overexpressed in metastatic lymph nodes of ductal carcinomas (103, 104). These findings suggest that, in later stages, connexins facilitate the metastatic involvement of locoregional lymph nodes. However, further studies are required to support this hypothesis.

Immunoregulatory cytokines, such as IL10, are important actors in tumor microenvironment associated with breast cancer. Specifically, IL10 is a pleiotropic anti-inflammatory cytokine with a dual role in breast cancer, exhibiting both pro- and anti-tumor activities (105). Its intricate molecular pattern of interactions has not been fully elucidated yet, however, this regulatory molecule is thought to take part in tumor initiation and progression, promoting immunosuppression and tumor immune evasion. IL10 predominantly displays a tumor-inhibiting activity through the activation of NK cells, enhancement in surface expression of MHC antigen and promoting tumor infiltration by neutrophil and macrophages (106). In the opposite way, IL10 may also reduce immune response against cancer, mainly decreasing the antigen presentation capacity and modulating the production of several cytokines. Hence, higher levels of IL10 may increase tumor immune escape and this hypothesis is consistent with the observation of increased IL10 concentration in serum of breast cancer patients, particularly in case of metastatic disease (89). Hypothesizing that gene amplification leads to an increase in protein expression, IL10 immunosuppressive properties could the metastatic potential of breast cancer, increasing the risk of lymph node involvement, which represents a well-known predisposing factor for BCLR. These assumptions on the possible prognostic value of IL10 amplification for lymphedema risk prediction remain largely speculative. However, some studies found higher IL10 levels in metastatic lymph nodes and IL10 polymorphisms associated with increased expression in patients with lymph node-positive breast cancer (107, 108). Interestingly, high IL10 levels were also found in inflammatory breast cancer, a particularly aggressive and highly metastatic form of breast cancer, in which this cytokine correlates with the presence of lymphovascular invasion (109). This parameter has been recently associated with an increased risk of BCRL in patients with left side localization.

In summary, there is no specific evidence to date that genetic alterations in primary tumor play a direct role in BCRL pathogenesis. However, the correlation between somatic mutations and higher rates of nodal involvement could indirectly lead to more aggressive therapeutic schemes, including ALND and axillary radiation, and thus increasing the odds of developing post-surgical lymphedema.

## Lymphangiogenesis-Related Mechanisms as Potentially Druggable Targets

All these novel data suggest that novel individualized therapeutic strategies can be realistically implemented. In particular, the crucial role of VEGF and the observation of BCRL improvement in patients treated with anti-VEGF monotherapy provided evidence for the possible role of anti-angiogenic drugs in lymphedema treatment (110). In particular, a pilot study was conducted in order to evaluate the efficacy and safety of bevacizumab, a monoclonal antibody directed against VEGF, in patients with lymphedema following breast cancer treatment (110). The working hypothesis was that VEGF-inhibitors could significantly reduce interstitial fluid collection through the modulation of vascular permeability, resulting in an indirect improvement of lymphatic obstruction and drainage. Preliminary study results confirmed the hypothesis that Bevacizumab has a role in interstitial fluid pressure and extracellular fluid volume reduction (NCT00318513). However, many aspects limit its use in clinical practice for breast cancer patients. To date, Bevacizumab is no longer approved for breast cancer treatment and there is only partial evidence regarding the use of VEGF-inhibitors in subjects without active cancer. Lymph fluid collection represents the starting point of BCRL, which is worsened by chronic inflammatory tissue response to protein-rich fluid accumulation. The modulation of immune signal molecules, such as interleukins, could reduce inflammation and tissue reaction, preventing lymphedema chronicization. In this setting, a trial is ongoing to test the efficacy of peripheral intravenous injections of a combination of two monoclonal antibodies that neutralize the biologic activity of IL4 and IL13 (NCT02494206). Further clinical studies are needed to develop targeted therapies directed to improve lymphatic regeneration and function, together with the modulation of inflammatory pathways. An appropriate medical treatment combining physical and molecularly targeted drugs administered early on after surgery in high-risk individuals could become the key strategy to prevent lymphedema formation.

## Conclusions

BCRL is a complex and underdiagnosed condition, with potentially devastating consequences on the quality of life of breast cancer survivors. Several genetic, anatomical, biological, and clinical factors might intervene in its development, supporting the hypothesis of a multifactorial etiopathogenesis. Impairment of the lymphatic system embryogenetic differentiation mechanisms, anatomical variations, alterations of the lymphatic pacemaking system, mechanisms of phasic contractions of the lymphatic vessel, and systemic inflammation might act synergistically. In addition, mutations in genes encoding inter-cellular communication have been linked to both primary and secondary lymphedema. There is no evidence that genetic alterations related to the different molecular subtypes of breast cancer could influence BCRL pathogenesis. On the other hand, medical, surgical, and radiation therapies are crucial factors in its development and progression. Further research is needed in order to clarify, according to a novel multidisciplinary approach, the strict correlation between clinical and biological aspects of BCRL. The identification of specific molecular targets, novel biomarkers, and validated risk stratification tools could prove significantly crucial, bringing us closer to achieving the goal of precision medicine for BCRL.

## Author Contributions

MI and NF: study concept and design. MI, RB, and NF: supervision. GL, AM, AS, and LR: manuscript writing (first draft). AM: bibliography. GL, KV, and ES: iconography. LD and MG: first draft revision. All authors: revision and approval of the final draft.

### Conflict of Interest

The authors declare that the research was conducted in the absence of any commercial or financial relationships that could be construed as a potential conflict of interest.
